# Electrocardiographic abnormalities in medically screened German military aircrew

**DOI:** 10.1186/s12995-021-00327-x

**Published:** 2021-08-31

**Authors:** Norbert Guettler, Stefan Sammito

**Affiliations:** 1 German Air Force Centre of Aerospace Medicine, Cologne, Germany; 2 Department of Cardiology, Justus Liebig University Giessen, University Hospital Giessen, Medical Clinic I, Giessen, Germany; 3 Department of Occupational Medicine, Medical Faculty, Otto von Guericke University, Magdeburg, Germany

**Keywords:** ECG screening, Pilot applicants, Aircrew, Prevention, Aerospace medicine

## Abstract

**Background:**

A resting electrocardiogram (ECG) is a well-tolerated, non-invasive, and inexpensive test for overt electrical signs of cardiac pathology and is widely used in the screening of aircrew and other high-hazard occupations. Given the low number of pathological results leading to disqualification or restriction however, there is an ongoing debate as to how often screening ECGs should be performed in different age groups.

**Methods:**

We restrospectively analyzed 8275 resting 12-lead ECGs registered between 2007 and 2020 in the German Air Force Centre of Aerospace Medicine. Findings were categorized according to consensus recommendations published by the NATO Working Group on Occupational Cardiology in Military Aircrew, based on ECG screening criteria published for athletes which were used at the time of registration. Age, sex, height, weight, and body mass index of the probands were also captured. Additionally, 4839 pilot and non-pilot aircrew members were analyzed longitudinally over a maximum period of 13.4 years.

**Results:**

Out of all the ECGs only 18 revealed findings requiring further investigation, and only one individual was temporarily disqualified because of a ventricular pre-excitation (delta wave) as a sign of an antegrade conducting accessory pathway. The longitudinal analysis of 25,829 ECGs revealed 28 abnormalities requiring further investigation, and only two ECG findings (in probands aged 48.8 and 59.1 years) led to temporary, or permanent disqualification. In a third case, the ECG showed signs of a myocardial infarction, which was already known from the proband’s history.

**Conclusions:**

Initial ECG screening for asymptomatic aircrew revealed extremely low numbers of individuals requiring further investigation in our cohort. This would appear to justify an initial screening ECG and follow-up ECGs at certain intervals starting at a certain age, but routine ECG screening of applicants in professions with a higher risk tolerance or frequent, e.g. annual, follow-up ECGs in younger aircrew is not supported by our data because of the minimal yield of ECG findings requiring further investigation.

## Background

A resting electrocardiogram (ECG) is a well-tolerated, non-invasive and inexpensive test for overt electrical signs of cardiac pathology. It is widely used in the screening of initial pilot applicants, as part of periodic medical examinations (PME) of trained aircrew, and in the screening of individuals applying for, or working in, high-hazard employment. According to the regulations of many civilian aviation licensing authorities, a 12-lead resting ECG is the only routinely performed machine-aided cardiological examination in a flight physical exam [1–4].

ECG screening of young individuals mainly concentrates on the detection of inherited channelopathies, delta waves, or signs of cardiomyopathies, whereas in individuals aged 40 years and over, it focuses on the diagnosis of coronary artery disease.

It has been shown in several studies that the proportion of abnormal ECG results in the screening of young, asymptomatic, and apparently healthy individuals is exceptionally low, and that many of these abnormal results can be regarded as “normal variants”, for example caused by a high vagal tone [2, 5–7]. This has led to an ongoing discussion about if, and how often, a screening ECG should be performed in young individuals, with the inherent risk of false positive results, potentially leading to fruitless investigations and subsequent social and economic costs. Labeling individuals with an uncertain diagnosis originating from a false-positive ECG finding can also lead to psychological effects, as well as possible impacts on insurance policies and employment.

All German military pilot applicants are aeromedically screened in the German Air Force Centre of Aerospace Medicine (GAFCAM) in Fuerstenfeldbruck, Germany. In addition, screening is also performed for licensed non-pilot aircrew. The initial screening includes a 12-lead resting ECG for both groups. After the initial screening German Air Force, Army and Navy pilots, as well as weapon system officers on fast jets, undergo PME at the same centre.

In this study, we analyzed 8275 screening ECGs from initial examinations of pilot and non-pilot applicants, as well as 4839 screening ECGs from PMEs of active service pilot and non-pilot aircrew over a period of maximum 13.4 years (2007–2020). Abnormal ECG results were analyzed for their relevance in an aeromedical context. The aim of this study was to retrospectively evaluate the incidence of positive results in aeromedical ECG screenings, the potential false-positive rate, and to determine appropriate ECG examination intervals.

## Methods

We analyzed data from initial examinations and PME in the GAFCAM between February 2007 and June 2020. Weapon system officers on fast jets are classed as pilots. Examination intervals are 3 years for pilots up to 40 years of age, with annual examinations by the local flight surgeons in the intervening years. Pilots above 40 years of age are examined annually at the GAFCAM. Licensed non-pilot aircrew are examined, with an ECG, annually by the local flight surgeon regardless of their age. Exceptions to this rule are non-pilot aircrew with a waiver according to the German waiver system, and flight surgeons. These two groups usually undergo annual examinations at the GAFCAM.

A total of 8275 ECGs registered during initial screening were retrospectively analyzed, 6284 of pilot applicants and 1991 of non-pilot aircrew applicants. For the longitudinal analysis, a total of 25,829 ECGs from 4839 aircrew (4216 pilots and 623 non-pilot aircrew) were captured. The custo card m™ ECG device (custo med GmbH, Ottobrunn, Germany) was used. ECG registration lasted for 4.4 s, the sweep speed was routinely 50 mm/s. The intervals (P wave, PQ interval, QRS complex, QT interval, QTc, and RR interval) were measured automatically, the corrected QT interval QTc was calculated by the device using Bazett’s formula: QT / √R-R interval, but all ECGs were also over-read and interpreted by one of the physicians working in the Department of Internal Medicine. Age, sex, height, weight, and body mass index (BMI) were also captured.

The discrepancy in numbers between aircrew applicants and active aircrew is due to the following reasons:

1. Not all aircrew applicants were accepted for aircrew duties.
2. Non-pilot aircrew only underwent their initial examination at the GAFCAM, follow-up examinations were performed by the local flight surgeon. Exemptions from this rule were aircrew with medical problems in any speciality, and flight surgeons who are categorically examined at the GAFCAM.
3. Some aircrew included in the longitudinal analysis had their initial examination prior to the start of the analysis period in 2007, when the institute information system changed, and data collection became possible.

Abnormal ECG results were retrospectively categorized into normal variants, those requiring further investigation, and those disqualifying for aircrew duties, according to the recently published consensus recommendations by the NATO Human Factor and Medicine (HFM)-251 Occupational Cardiology in Military Aircrew working group (Table 1) [1]. The ECG criteria for aircrew published in 2019 were based on several publications on ECG screening criteria for athletes which were used for ECG evaluation at the time of registration [8–10]. Although there are differences between athletes and aircrew concerning the intensity of training, ECG evaluations based on those criteria were practically almost identical to the retrospective evaluation based on the 2019 criteria for aircrew. Further investigations triggered by abnormal ECG findings and their outcomes were also analyzed.

**Table 1:** Recommendations for the assessment of ECG findings in aircrew [1]. BMI = body mass index; QTc = corrected QT-interval; yr = years; bpm = beats per minute

Normal ECG variants	For normal variants, consider further investigation if…	ECG findings requiring further investigation	ECG findings disqualifying for high-risk duties unless treated, with resulting acceptable risk
Sinus arrhythmia	Sino-atrial block (< 3 s/day, < 4 s/night)	Sinus arrest Sino-atrial block > 3 s/day, > 4 s/night	
Ectopic atrial rhythm with inverted P waves in the inferior limb leads	Symptoms suggestive of tachycardia		
Sinus bradycardia ≥40 bpm	Sinus bradycardia < 40 bpm Junctional rhythm	Idioventricular rhythm	
Sinus tachycardia > 100 bpm	Persistent sinus tachycardia > 100 bpm at rest		
1st degree AV block – PR < 300 ms		1st degree AV block – PR > 300 ms	
2nd degree AV block Mobitz I (Wenckebach)	first appearance at age > 40 yr or if frequent, especially while awake		2nd degree AV block Mobitz II 3rd degree AV block
Incomplete right bundle branch block Left anterior fascicular block	New Left anterior fascicular blockblock > 40 yr	Complete right bundle branch block Left bundle branch block Left posterior fascicular block	
Single premature atrial complex (PAC) or premature junctional complex (PJC)		> 1 premature atrial complex (PAC) or premature junctional complex (PJC) Supraventricular tachycardia < 30 s	Supraventricular tachycardia > 30 s or symptomatic
Single premature ventricular complex (PVC)		> 1 premature ventricular complex (PVC) or ≥ 1 pair Ventricular tachycardia ≤11 beats	Ventricular tachycardia > 11 beats or symptomatic
Short PR interval 90-120 ms with no evidence of delta waves	Very short PR < 90 ms	asymptomatic ventricular pre-excitation	Wolff-Parkinson-White (WPW) syndrome
Isolated QRS voltage criteria for left ventricular hypertrophy, especially in young people	Elevated BP, BMI > 30, age > 40 yr, or new finding	Left ventricular hypertrophy with strain	
Atrial enlargement	Accompanied by axis deviation		
Right ventricular hypertrophy (R wave in V_1_ plus S wave in V_5_ or V_6_ > 10.5 mm)			
ST segment depression and/or negative T wave only in lead III		Diffuse T wave abnormality or ST changes	
QTc prolongation (QTc < 470 ms)	Family history of long QT syndrome	QTc > 470 ms but < 500 ms	QTc > 500 ms
Early repolarization (benign form) - no evidence of delta waves		Brugada Type 2	Brugada Type 1 pattern

Statistical analyses were conducted using IBM SPSS Statistics for Windows 24 (IBM Corp. Released 2016, Armonk, NY, USA). Data analysis was primarily descriptive. Because the Kolmogorov-Smirnov test showed that none of the nominal scale parameters was normally distributed, median, and interquartile range (IQR) were calculated. Differences were analyzed with Pearson’s chi-squared test, and for independent samples Mann-Whitney U test was used. Significance level was defined as *p* < 0.05.

According to the regulations of the Bavarian Medical Association, the responsible authority for this study, a vote of the ethics committee was not necessary for this retrospective analysis without a risk to the participants. All data was analyzed as pseudonymized records.

## Results

Baseline characteristics of the included applicants are illustrated in Table 2. Average age, BMI, weight, and the percentage of females were higher, and the height was lower in non-pilot aircrew than in pilot aircrew applicants (*p* < 0.001).

**Table 2:** Baseline characteristics of the included applicants for pilot and non-pilot aircrew. Age, height, weight, and BMI are given as median (Interquartile Range (IQR); 25–75%); BMI = body mass index

	Pilot aircrew	Non-pilot aircrew
**Number (n)**	6284	1991
**Age (years)**	20.0 (3.0)	28.4 (9.9)
**Sex**		
**male, n (%)**	6092 (96.9)	1564 (78.6)
**female, n (%)**	192 (3.1)	427 (21.4)
**Height (cm)**	180.3 (8.9)	178.4 (11.0)
**Weight (kg)**	75.0 (13.4)	79.3 (17.4)
**BMI (kg/cm**^**2**^**)**	23.0 (3.5)	24.8 (4.4)

**Fig. 1:**
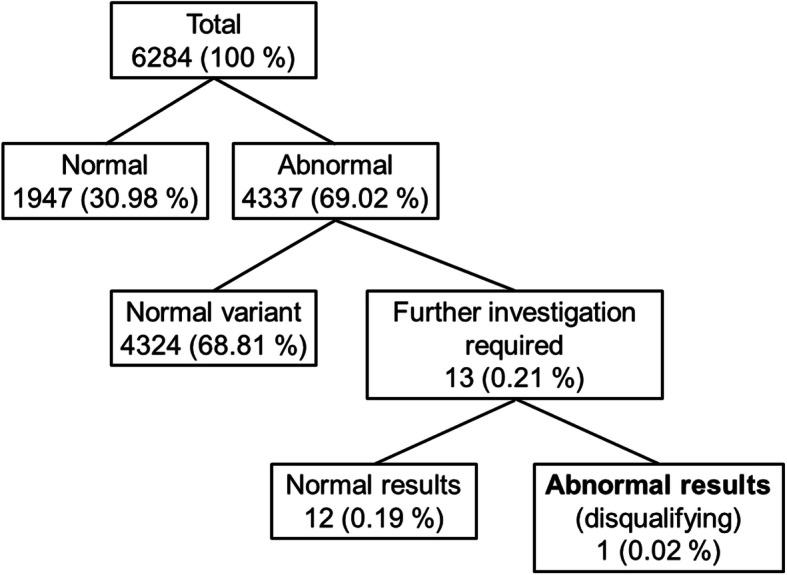
Classification of ECG findings in pilot applicants

There was no difference between the results of the original ECG analysis at the time of registration and those based on the criteria published in 2019 [1]. Table 3 shows all ECG findings seen in pilot and non-pilot applicants. These findings are attributed to applicants, so one proband may have a combination of two or three abnormal findings. The probands are categorized into those with findings regarded as normal variants without the need for further investigation, and those requiring further investigation (see Fig. 1).

**Table 3:** ECG findings in pilot and non-pilot aircrew applicants from the initial examination (ordered by the need for further investigation). The total number of findings exceeds the number of applicants, because one applicant may be affected by more than one finding. Percentages are calculated in relation to the number of applicants. AV = atrioventricular; RBBB = right bundle branch block; HR = heart rate; bpm = beats per minute; ECG = electrocardiogram; *n* = number. * = pilot applicants routinely undergo transthoracic echocardiography. None of these revealed left ventricular hypertrophy

	pilots		non-pilot aircrew	Further investigation required	
Abnormal finding(s)	n	%	n	%	
Complete RBBB	–	–	1	0.05	**Yes**
Diffuse ST / T segment changes	1	0.02	–	–	**Yes**
Sinus bradycardia (HR < 40 bpm)	7	0.10	3	0.15	**Yes**
ST segment and T wave abnormalities II, III, aVF	3	0.05	–	–	**Yes**
Suspected delta wave	–	–	1	0.05	**Yes**
Ventricular pre-excitation (delta wave)	1	0.02	–	–	**Yes**
1 premature atrial or ventricular complex	117	1.86	18	0.90	No
1st degree AV Block (PR < 300 ms)	2	0.03	–	–	No
Benign early repolarization	3	0.05	–	–	No
Discrete ST / T segment changes^1^	2491	39.64	303	15.22	No
Ectopic atrial rhythm	2	0.03	–	–	No
Incomplete RBBB	10	0.16	1	0.05	No
Intermittent junctional rhythm	1	0.02	–	–	No
Left anterior fascicular block (age < 40 years)	2	0.03	1	0.05	No
Negative T wave in III	5	0.08	4	0.20	No
QRS voltage criteria for left ventricular hypertrophy*	9	0.14	2	0.10	No
Sinus bradycardia (HR ≥ 40 and < 60 bpm)	1551	24.68	492	24.71	No
Sinus tachycardia (HR > 100 bpm, not persistent at rest during physical examination)	123	1.96	31	1.56	No
Slightly broadened QRS complexes (133 ms)	1	0.02	–	–	No
Normal ECG	1959	31.17	1136	57.06	No
**Total number of applicants**	**6284**	**100**	**1991**	**100**	

^1^ slight ST segment depressions of < 0.5 mm in depth, flat T waves or slight T wave inversions of < 1 mm in depth

6284 ECGs of pilot applicants were assessed, 1947 of which were classified as entirely normal. 4337 ECGs presented abnormal findings described by the assessing physician, but only 13 (0.21%) required further investigation. The remainder were deemed as acceptable variants. One of the truly abnormal ECGs revealed a ventricular pre-excitation (delta wave) as a sign of an antegrade conducting accessory pathway. According to the aeromedical regulations of the German Armed Forces, this finding requires an invasive electrophysiological testing for further risk stratification, so this applicant was primarily assessed as unfit for flying duties. In the other twelve cases, the cardiological evaluation did not reveal any abnormal result which indicated underlying cardiac disease.

**Fig. 2:**
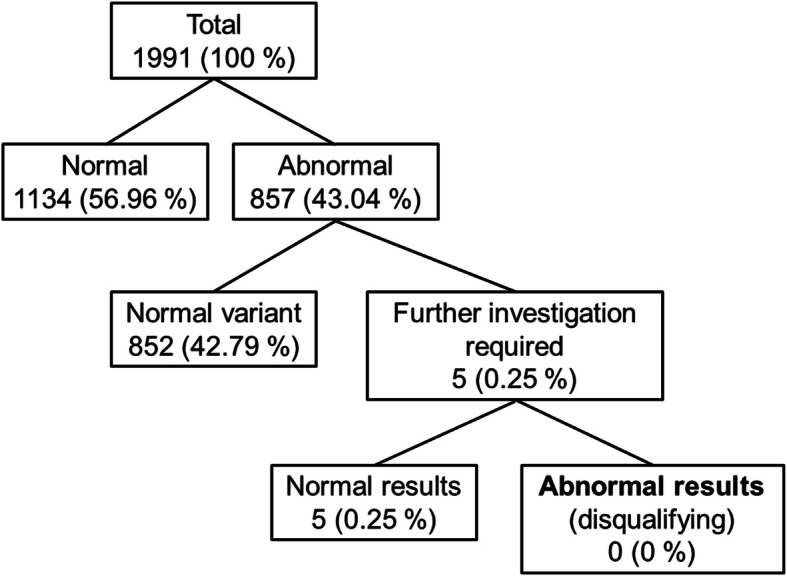
Classification of ECG findings in non-pilot aircrew applicants

Out of 1991 ECGs of non-pilot aircrew applicants, 1134 were assessed as normal, whilst 857 revealed abnormal results. Only five of these required further investigations which all showed normal results (see Fig. 2).

Further cardiological investigation of pilot and non-pilot aircrew applicants with abnormal resting ECG results included an exercise ECG in all cases, an echocardiogram in 14 cases, and Holter monitoring in 2 cases. In one non-pilot aircrew applicant a delta wave was identified, but an antegrade conducting accessory pathway was excluded by adenosine testing.

For the longitudinal analysis of pilots and non-pilot aircrew (*n* = 4839; initial age 30.1 years (IQR 18.8 years); median follow-up period 6.7 years (1 to 13.4 years)) a total of 25,829 resting ECGs were analyzed. We observed a change from a normal to an abnormal ECG requiring further investigation in only 28 individuals (0.6%) (Table 4). The median age of these probands was 44.4 years (IQR 16.0 years), when the abnormality was detected. Only two of these findings disqualified the individuals from flying duties; one was a single episode of atrial fibrillation with tachyarrhythmia (detected at 48.8 years of age), and the other, a loss of R waves in the precordial leads caused by a myocardial infarction (detected at 59.1 years of age). This myocardial infarction was not previously known and was diagnosed by computed tomography (CT) coronary angiography because of the ECG finding. Whilst the first proband sebsequently returned to unrestricted flying duties (having had a single episode of atrial fibrillation caused by an infection), the second proband was disqualified from flying for the rest of his career. Complete right bundle branch block in one proband had been known for many years, a coronary artery disease had been excluded. The proband had a mild medically treated hypertension and underwent regular follow-ups with echocardiography and exercise ECG.

**Table 4:** ECG changes in pilots and non-pilot aircrew over a longitudinal period of 13.4 years. HR = heart rate; bpm = beats per minute; *n* = number; * = This myocardial infarction was already known from the patient’s history; the proband would have been disqualified regardless of his ECG findings

ECG abnormality	N	Disqualification because of ECG finding
Intermittent ventricular pre-excitation, known from medical history	1	No
Single episode of atrial fibrillation with tachyarrhythmia	1	**Yes**
QRS voltage criteria for left ventricular hypertrophy	2	No
Escape beats with left bundle branch block morphology (one single episode)	1	No
Previous myocardial infarction	1	No*
Complete right bundle branch block (no progression from incomplete block)	1	No
Loss of R waves in precordial leads	1	**Yes**
Sinus bradycardia (< 40 bpm)	17	No
Negative T waves	3	No
**Total**	**28**	

## Discussion

This is, to the best of our knowledge, the largest analysis of screening ECGs of initial applicants for aircrew duties and of active aircrew members. The study retrospectively analyzed 8275 resting 12-lead ECGs of initial applicants for aircrew duties and 25,829 ECGs of active-duty aircrew in the German Air Force, Army and Navy, registered between 2007 and 2020. In summary, this analysis shows that only very few applicants show abnormal ECGs disqualifying for aircrew duties. Screening ECGs of active aircrew also revealed very few disqualifying results registered in aircrew of advanced age.

The use of ECG screening is commonplace not only in aviation medicine but also in other high-hazard occupations such as law-enforcement, commercial diving, offshore working, fire fighting, and professional driving. Additionally, it is used for screening of professional athletes [11, 12]. In the occupational setting of aircrew, failing to identify silent cardiac disease may have catastrophic consequences. International recommendations for cardiological screening and ECG interpretation have therefore been published [1]. Criteria for normal and abnormal ECG changes were taken from guidelines and literature on ECG interpretation, but they are often based on the Seattle criteria for athletes [8–10]. According to these criteria, some ECG changes can be regarded as normal variants in young people because of their training, whilst others require further examination. The difference found between pilot and non-pilot applicants (Table 3) cannot be fully explained. It may be due to the younger average age, and/or better cardiopulmonary fitness and training condition in pilot applicants.

There are different requirements for ECG screening between civilian licensing authorities. The European Union, for example, requires ECG screening for a class 1 medical certificate (professional pilots), at the initial examination, then every 5 years until age 30, every 2 years until age 40, annually until age 50, and at all revalidation or renewal examinations thereafter. For a class 2 medical certificate (private pilots), it has to be carried out at the initial examination, at the first examination after age 40 and then at the first examination after age 50, and every 2 years thereafter [3]. According to the regulations issued by the Federal Aviation Administration (FAA) in the US, a 12-lead resting ECG is required for first-class medical certification (airline transport pilot) at the first application after reaching the 35th birthday, on an annual basis after reaching the 40th birthday, and on a clinical indication. Without a clinical indication, second-class (commercial pilot) and third-class (private pilot) medical certification does not require a routine ECG [4].

Although most screening ECGs in asymptomatic individuals show normal results or likely normal physiological changes, there is a minority of individuals with ECG changes that are suggestive of genuine cardiac pathology, that may lead to disqualification or restriction [5, 13, 14]. False-positive results from ECG screening, however, should be minimized, as they may lead to unnecessary, time-consuming, and costly downstream, evaluations. Additionally, labelling individuals with an uncertain diagnosis originating from a false-positive ECG finding can lead to psychological effects, as well as possible impact on insurance policies and employment. To avoid misinterpretation of screening ECGs, the published standardized criteria should strictly be followed [1, 9].

In our cohort, the prevalence of abnormal ECG findings requiring further investigation in applicants was comparatively low at 0.21 and 0.25% respectively, in pilots and non-pilot aircrew. Only a fraction of these findings led to disqualification from aircrew duties. However, civilian applicants for a military pilot career, as well as active soldiers applying for aircrew duties, are pre-selected personnel. They must undergo a basic medical examination, which does not include an ECG, but may exclude applicants with a cardiac disease by means of medical history and physical examination. Other studies evaluating the prevalence of abnormal ECG screening results in young and healthy soldiers found a prevalence between 0.6 and 7.0% [14, 15]. In a group of 32,652 young athletes (median age 17 years) undergoing pre-participation ECG screening, the prevalence of abnormal results was 11.8% [16]. Electrocardiographic parameters vary across different ethnicities and in comparison with international norms [15, 17]. It can be assumed that the prevalence of abnormal ECG results in our study was reduced by the basic medical examination prior to the aeromedical assessment in our institution. However, the detected abnormal results, such as the ventricular pre-excitation (delta wave), show that resting ECG screening for this high risk occupation can detect abnormalities that are likely to increase the risk of a sudden incapacitation in flight or other catastrophic events [18].

As in applicants for aircrew duties, screening ECGs of active pilot and non-pilot aircrew also revealed a low percentage of abnormalities that led to disqualification from aircrew duties (0.6%). This may be because aircrew are preselected personnel having passed the initial examination. They also have a distinctive health awareness because of the continuous health education by the local flight surgeon and the GAFCAM, and because of their annual PMEs undertaken throughout their whole career. In the very few cases with disqualifying abnormalities in our cohort the ECG turned out to be a useful tool, which possibly prevented sudden incapacitation in flight. These abnormalities, however, were seen in aircrew of nearly 50 and nearly 60 years of age, respectively.

There is also a risk of false-negative ECG results, for example if cardiac pathology does not cause an ECG abnormality, such as premature coronary artery disease or anomalous coronary anatomy. Other examples that may not be detected on resting 12-lead ECGs include inherited cardiac conditions, that have not developed sufficiently to show an abnormal ECG; ECG abnormalities presenting intermittently or on provocation, such as borderline QT prolongation or Brugada ECG; or ECG abnormalities that are not seen at rest, e.g., rate related bundle branch block. It is generally difficult to verify false-negative ECG results; and with the data in our study, it is impossible. However, there have been a number of autopsy studies in which coronary artery disease was detected in pilots post mortem, which was previously undetected [19, 20].

It is also very difficult to perform a cost benefit analysis that includes false-negative ECG results. However, due to its low sensitivity and specificity, it has been calculated that each air accident prevented by ECG screening costs over 100 million euro [13, 21]. Despite of this poor cost benefit relationship, many authors argue for resting ECG screening because it has been shown to be more sensitive in detecting cardiovascular disease than medical history or physical examination either alone or in combination, and because sudden incapacitation and/or sudden cardiac death of a pilot commanding an aircraft is a devastating event [14].

Based on our assessment, it may be reasonable to perform an ECG at the initial examination at least of pilots, then for all aircrew at the age of 40 years, 45 years, and 50 years, and every 2 years thereafter. As in other studies [14–17] the proportion of abnormal findings in screening ECGs was higher than in our cohort, significantly more data would be needed to completely abandon initial screening ECGs in young pilot applicants.

The presented study has strengths and some limitations. One of the strengths is the comprehensive analysis of a large sample of resting ECG results over a long period of time. The examinations were performed under constant and standardized conditions. As a 12-lead resting ECG is an obligatory part of every aeromedical assessment for aircrew, every single proband was captured. An additional strength of the study was the measurement of ECG intervals and heart rate leading to the detection of ECG changes, which in our cohort were regarded as normal variants and did not cause disqualification or restriction.

A limitation of this study is the bias that is inherent in the preselection of the applicants by a basic medical examination prior to aeromedical assessment. This preselection, mainly consisting of a medical history and a basic physical examination, may have reduced the prevalence of abnormal results compared to other studies. It may be assumed, however, that individuals with known cardiovascular diseases would probably not apply for a career as a military pilot. This kind of preselection may therefore be typical for aeromedical assessment. One additional important limitation of our study may be the fact that ECG intervals were measured automatically, but the overall ECG interpretation had to be done by the AME. Although all the AMEs were experienced in ECG interpretation, their skills and experience might have been variable. Modern computerized algorithms for ECG assessment might be useful to obtain objective results. The accuracy and plausibility of these automated results, however, should be verified by an experienced physician.

## Conclusions

Our retrospective analysis of screening ECGs from pilot and non-pilot aircrew applicants as well as the longitudinal analysis of screening ECGs from active pilot and non-pilot aircrew over a maximum period of 13.4 years revealed a very low yield of abnormal findings requiring further investigation or even disqualification for aircrew duties. Despite the low prevalence of ECG findings indicating silent cardiac disease, certain high-hazard occupations with a very low risk tolerance, including pilots, justify initial ECG screening given the potentially disastrous consequences of failing to identify cardiac conditions that may potentially lead to sudden incapacitation or even sudden cardiac death.

However, our study suggests that relevant abnormalities in follow-up ECGs are rare and occur particularly in older pilots. This would appear to justify screening ECGs at certain intervals, starting at a certain age (e.g., above 40), but regular routine ECG screening of applicants in professions with a higher risk tolerance or frequent, e.g., annual follow-up ECGs in younger aircrew is not supported by our data.

## Data Availability

The data that support the findings of this study are available from the Federal Ministry of Defense. Data are available on reasonable request.

## Abbreviations

AV: Atrioventricular
BMI: Body mass index
bpm: Beats per minute
CT: Computed tomography
ECG: Electrocardiogram
FAA: Federal aviation administration
GAFCAM: German air force centre of aerospace medicine
HFM: Human factor and medicine
HR: Heart rate
ICR: Interquartile range
n: Number
PME: Periodic medical examination
QTc: Corrected QT-interval
RBBB: Right bundle branch block
yr: Years
